# Distribution of protein poly(ADP-ribosyl)ation systems across all domains of life

**DOI:** 10.1016/j.dnarep.2014.05.003

**Published:** 2014-11

**Authors:** Dragutin Perina, Andreja Mikoč, Josip Ahel, Helena Ćetković, Roko Žaja, Ivan Ahel

**Affiliations:** a Division of Molecular Biology, Ruđer Bošković Institute, Zagreb 10002, Croatia; b Division for Marine and Environmental Research, Ruđer Bošković Institute, Zagreb 10002, Croatia; c Sir William Dunn School of Pathology, University of Oxford, South Parks Road, Oxford OX1 3RE, UK

**Keywords:** ADPr, ADP-ribose, PAR, poly(ADP-ribose), PARP, poly(ADP-ribose) polymerase, PARG, poly(ADP-ribose) glycohydrolase, TARG1, terminal ADP-ribose protein glycohydrolase, ARH, ADP-ribosylhydrolase, Poly(ADP-ribose), PARP, PARG, Macrodomain, DNA damage response

## Abstract

Poly(ADP-ribosyl)ation is a post-translational modification of proteins involved in regulation of many cellular pathways. Poly(ADP-ribose) (PAR) consists of chains of repeating ADP-ribose nucleotide units and is synthesized by the family of enzymes called poly(ADP-ribose) polymerases (PARPs). This modification can be removed by the hydrolytic action of poly(ADP-ribose) glycohydrolase (PARG) and ADP-ribosylhydrolase 3 (ARH3). Hydrolytic activity of macrodomain proteins (MacroD1, MacroD2 and TARG1) is responsible for the removal of terminal ADP-ribose unit and for complete reversion of protein ADP-ribosylation.

Poly(ADP-ribosyl)ation is widely utilized in eukaryotes and PARPs are present in representatives from all six major eukaryotic supergroups, with only a small number of eukaryotic species that do not possess PARP genes. The last common ancestor of all eukaryotes possessed at least five types of PARP proteins that include both mono and poly(ADP-ribosyl) transferases. Distribution of PARGs strictly follows the distribution of PARP proteins in eukaryotic species. At least one of the macrodomain proteins that hydrolyse terminal ADP-ribose is also always present. Therefore, we can presume that the last common ancestor of all eukaryotes possessed a fully functional and reversible PAR metabolism and that PAR signalling provided the conditions essential for survival of the ancestral eukaryote in its ancient environment.

PARP proteins are far less prevalent in bacteria and were probably gained through horizontal gene transfer. Only eleven bacterial species possess all proteins essential for a functional PAR metabolism, although it is not known whether PAR metabolism is truly functional in bacteria. Several dsDNA viruses also possess PARP homologues, while no PARP proteins have been identified in any archaeal genome.

Our analysis of the distribution of enzymes involved in PAR metabolism provides insight into the evolution of these important signalling systems, as well as providing the basis for selection of the appropriate genetic model organisms to study the physiology of the specific human PARP proteins.

## Highlights

• PARPs are present in representatives from all six major eukaryotic supergroups.
• Reversible PAR metabolism was established early in eukaryotic evolution.
• The last common ancestor of all eukaryotes possessed five types of PARP proteins.
• PARPs are associated to a large variety of different pathways.

## 1 Introduction

Poly(ADP-ribosyl)ation is a post-translational modification of proteins in which multiple ADP-ribose nucleotide moieties are transferred to specific target proteins forming poly(ADP-ribose) (PAR) chains. Poly(ADP-ribosyl)ation can alter the physical and chemical properties of target proteins and controls many important cellular processes such as DNA repair, transcription, regulation of centromere function, telomere length and ageing, protein degradation, apoptosis and necrosis [1], [2]. The only known proteins capable of poly(ADP-ribosyl)ation are members of poly(ADP-ribose) polymerase (PARP) family. PARPs are related to ADP-ribosylating bacterial toxins; they share the same protein fold and belong to the H-Y-E class of ADP-ribosyltransferase (H-Y-E denotes the catalytic triad His-Tyr-Glu) [3]. All PARPs catalyze the transfer of an ADP-ribose (ADPr) from NAD^+^ to target proteins [4] by covalently attaching ADPr to the glutamate or aspartate residues on the target proteins through an ester bond (protein mono(ADP-ribosyl)ation) [5], [6], [7]. Some PARP family members can attach subsequent ADPr units via 2′,1″ O-glycosidic ribose–ribose bonds to produce long linear chains of PAR. Occasional branching of PAR polymer may occur every 20–50 residues through 2″,1‴ O-glycosidic bond [4], [8].

The poly(ADP-ribosyl)ation of proteins is thought to inherently occur only in eukaryotes. Evolutionary analysis suggests that the eukaryotic PARP family can be subdivided into six clades based on phylogenetic analyses of PARP catalytic domains [9]. The human genome encodes 17 different PARPs, with different functions and belonging to five distinct clades. Clade 1 includes human PARP1, PARP2 and PARP3 enzymes. These PARPs are specifically involved in DNA break repair, chromatin regulation and transcription [10]. PARP1 is the best studied member of the PARP superfamily, with a well-defined and detailed structural basis for its DNA damage-dependent activity [11]. This protein consists of six domains: three Zn-binding domains, BRCT, WGR and PARP. PARP1 is responsible for the majority of PARP activity in the cell [12]. PARP1 and PARP2 possess both overlapping and non-redundant functions in the maintenance of genomic stability. The expression of both PARP1 and PARP2 and/or DNA-dependent poly(ADP-ribosyl)ation is essential during early embryogenesis in mice [13]. Homologues from representatives of four eukaryotic supergroups, as well as bacteria (see below), show the ability to be induced by DNA damage and are involved in functions related to DNA metabolism [14], [15], [16], [17], [18]. Besides human PARP1, PARP2 and PARP3 homologues, other Clade 1 representatives have also been found in a variety of organisms [9].

Clade 2 PARPs consists of plant PARPs with representatives found in bryophytes through to angiosperms [9], [19]. This clade includes proteins with plant-specific RST domain. Proteins of Clade 2 are involved in stress response and may also function in transcriptional regulation [20].

**Fig. 1:**
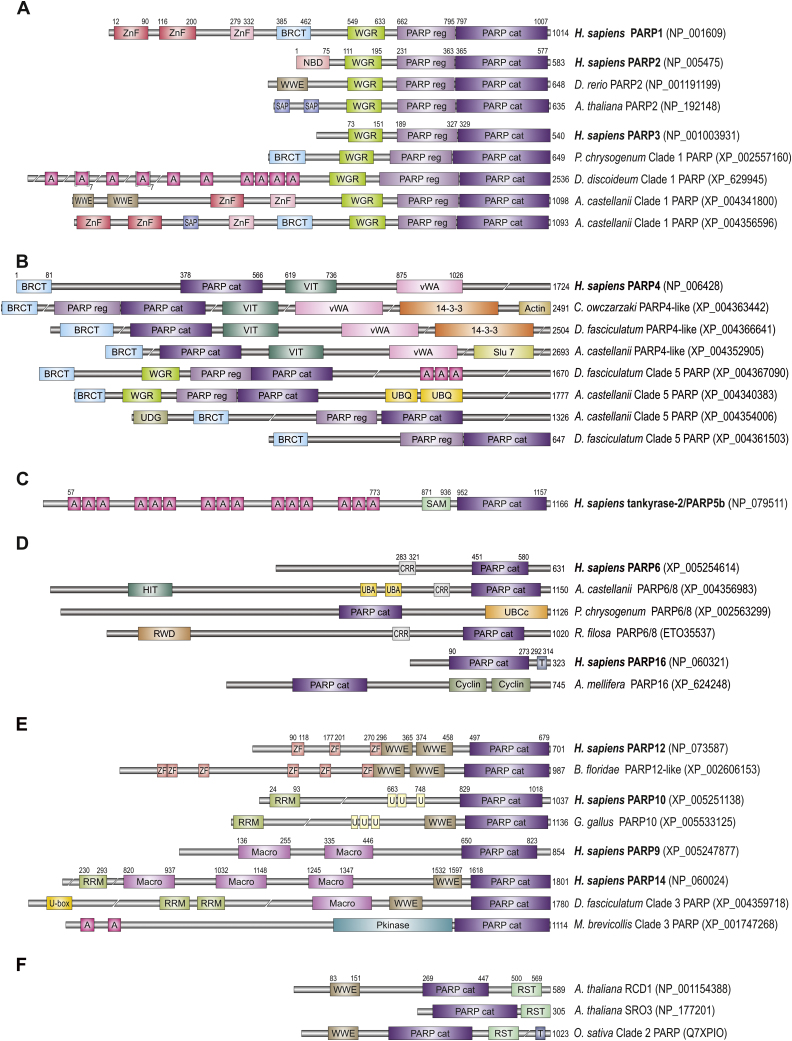
Schematic architecture of domains present in PARP representatives. PARPs belonging to six clades are assorted in six panels (A–E). Numbers indicate amino acids. Proteins are represented in a scale 1:10 (1 mm = 10 amino acids). Protein domains have been indicated with coloured boxes and each protein has been searched against SMART/Pfam databases. Abbreviations of domain names are retrieved from SMART/Pfam databases and indicated in figure. Shortened names include: ZnF (red and pink), DNA-binding zinc finger domains Zf-PARP and PADR1, respectively; A, Ankyrin (ANK); T, transmembrane region (TM); ZF, RNA-binding zinc finger ZnF_C3H1; U, ubiquitin-interacting motif (UIM); Domains which are not retrieved from SMART/Pfam databases: NBD, nucleic acid binding domain according to [60]. CRR, cysteine-rich region with putative zinc finger.

Clade 3 includes human PARP7 and PARP9-15, proteins that are heterogeneous both by their domain structure and function [9], [21]. For human PARP7, PARP10 and PARP14 mono(ADP-ribosyl) transferase activity has been suggested [22], [23]. PARP7 homologues are characterized by a ZnF_C3H1 zinc finger domain followed by a WWE domain and a PARP domain. Similar domain composition is present in PARP12 and PARP13 (Fig. 1E). WWE domains are often found in proteins involved in ADP-ribosylation and ubiquitinylation pathways [24]. The presence of ZnF_C3H1 in PARP7, PARP12 and PARP13 enables RNA-binding [25]. PARP11 homologues contain only WWE and PARP domains. Human PARP10 homologues contain an RRM domain followed by two ubiquitin-associated UIM domains and a PARP domain. The key functional domain, common to human PARP9, PARP14 and PARP15 proteins is the macrodomain, an ADP-ribose binding module [26] involved in diverse cellular processes such as DNA repair, chromatin remodelling and transcriptional regulation [27], [28], [29].

Tankyrases group in Clade 4 [9]. These enzymes function as poly(ADP-ribose) polymerases and are thought to have roles in controlling spindle formation during mitosis, the Wnt signalling pathway, proteasome assembly, vesicular trafficking and telomere maintenance [30], [31], [32], [33]. Human tankyrase 1 and tankyrase 2 are characterized by N-terminal ankyrin repeats with large capacity for protein–protein interactions, followed by a sterile alpha motif (SAM), important for multimerization of tankyrases, and PARP domain. Deficiency in both tankyrases in mice was shown to be embryonic lethal [34].

Clade 5 encompasses PARP4 (vPARP) homologues [9]. These proteins are associated with large ribonucleoprotein particles, located in the cytoplasm, named vaults. Beside VIT and vWA domains, which are usually found in tandem in proteins of multiprotein complexes associated with vaults, PARP4 proteins in animals also possess the BRCT domain usually found in proteins involved in DNA repair and cell cycle regulation [35]. Vaults have been implicated in the regulation of several cellular processes including transport mechanisms, signal transduction, immune responses and DNA repair [36]. The function of PARP4 in vaults is still unclear.

Human PARP6, PARP8 and PARP16 are predicted to act as mono(ADP-ribosyl) transferases and group together in Clade 6 [1], [9]. Non-catalytic domains of human PARP6 and PARP8 have not been characterized, whilst PARP16, in addition to a PARP domain, has a further transmembrane region at the C-terminal end. Human PARP16 protein also contains an α-helical domain [37]. Importance of this module is supported by its conservation from insects to humans [38]. In general however, members of Clade 6 are poorly characterized.

One representative of Clade 1 and one of Clade 6 were likely present in a common eukaryotic ancestor [9]. It was proposed that the ancestral Clade 1 member was structurally similar to recent human PARP2/3 and consisted of WGR, PARP regulatory and PARP catalytic domains, while the Clade 6 representative was likely similar to recent PARP6/8 [9]. Although five supergroups of eukaryotes contain sequenced representatives with PARP genes in their genomes some lineages appear to have lost all PARP homologues; i.e. in sequenced diatoms, brown algae, red algae, subset of green algae and Excavata group Diplomonads, PARP homologues were not previously identified [9]. The fungal lineage is most interesting from perspective of gene loss, where it is suggested that at least five independent losses of PARPs have occurred [9]. Consequently, yeasts do not have PARPs.

In non-eukaryotes, scattered PARPs acquired through horizontal gene transfer were found in several bacterial genomes. They encode catalytically active PARP orthologues with unknown function [2], [18]. The Archaea lack detectable PARP homologues although a thermoprotein with PARP-like activity from *Sulfolobus solfataricus* has been described [39]. PARP-like proteins were also found to be coded in the genomes of two double stranded DNA viruses [38].

The PAR modification of proteins needs to be reversed in order to regain their basal physiological functions. The main protein that hydrolyses poly(ADP-ribosyl)ation is poly(ADP-ribose) glycohydrolase (PARG). PARG deficiency is lethal in mouse and fruit fly, which indicates the critical importance of the PAR removal [40], [41]. PARG follows the phylogenetic distribution of PARPs and is found in all eukaryotes, with the exception of yeast. PARG uses the ADPr-binding macrodomain fold to specifically cleave PAR chains releasing the ADPr monomers [18], [42], [43]. Vertebrate PARGs contain regulatory and accessory domains that precede the PARG catalytic macrodomain [44]. The simplest, single-domain type of PARG (called bacterial-type PARG, bactPARG) is found in some bacteria and filamentous fungi [18]. Another possible mechanism of PAR hydrolysis is catalysis by ADP-ribosylhydrolase 3 (ARH3) which belongs to the dinitrogenase reductase-activating glycohydrolase-related protein family [45]. Neither PARG nor ARH3 are capable of efficient cleavage of the ester bond between the proximal ADPr unit and target proteins. Recent studies however have identified several other macrodomain-containing proteins that are capable of this reaction; specifically, human proteins called TARG1 (C6orf130), MacroD1 and MacroD2 were shown to be able to hydrolyze PARP-mediated protein mono(ADP-ribosyl)ation [2], [6], [46], [47]. These discoveries establish the complete reversibility of poly(ADP-ribosyl)ation as a regulatory modification. Macrodomains are widespread in all three domains of life and they can bind to different poly and mono(ADP-ribosyl)ated targets [48]. Besides macrodomains, another three evolutionary conserved PAR-binding modules have been described: PBM (PAR-binding motif) [49], PAR-binding zinc finger (PBZ) [50] and WWE domains [51].

In this paper we present the distribution and pattern of representation of proteins and domains involved in PAR metabolism across all domains of life. We show that the common ancestor of all eukaryotes possessed more PARP proteins than was previously thought. Since the distribution of PARPs follows the distribution of proteins capable of reversing PAR modification in the large majority of eukaryotic species we can presume that the last common ancestor of all eukaryotes possessed a fully functional and reversible PAR metabolism. The vast majority of recent eukaryotes maintained an active PAR metabolism and only several eukaryotic species adjusted to life without it. Only rare representatives from Bacteria possess all proteins required for active PARP metabolism.

## 2 Methods

The majority of sequences were obtained from NCBI non-redundant (NR) database using human protein sequences as a query (http://blast.ncbi.nlm.nih.gov/Blast.cgi). When sequences were not available in the NR database, BLASTP on Ensembl database (http://www.ensembl.org/index.html), TBLASTN on EST and WGS database on Genbank (http://www.ncbi.nlm.nih.gov/genbank/) were used. Additionally, genomes were searched at http://www.broadinstitute.org/annotation/genome/multicellularity_project/GenomesIndex.html, http://genome.jgi.doe.gov/and http://cyanophora.rutgers.edu/cyanophora/home.php. We focused on model organisms with fully sequenced genomes to avoid the possibility that some PARP proteins that are currently described as absent from specific organisms have simply not yet been identified. For example, the most recently sequenced genome used in our analyses (from Rhizaria species *Bigelowiella natans*) had 6.90× assembled sequence coverage and 98.9% of main genome in scaffolds >50 kB [52].

Domain architectures of retrieved sequences were obtained from the databases Pfam (http://www.sanger.ac.uk/Software/Pfam), SMART (http://smart.embl.de/) and PROSITE (http://prosite.expasy.org/) and examined through the NCBI conserved domain search website (http://www.ncbi.nlm.nih.gov/Structure/cdd/cdd.shtml). Secondary structure prediction was performed usingPhyre2 (http://www.sbg.bio.ic.ac.uk/phyre2/).

The collected amino acid sequences of PARP catalytic domains were aligned with MUSCLE3.8.31 multiple alignment tool, using default settings [53]. The multiple alignment was subjected to a maximum-likelihood (ML) analysis using MEGA5.2 [54]. The model for ML analysis was selected with ProtTest 2.4 [55] and the Akaike information criterion (AIC) [56], which indicated the General Reverse Transcriptase + Freq. model [57]. Bootstrap tests were performed with 1000 replicates.

## 3 Results and discussion

PARPs are the only family of enzymes capable of synthesis of PAR and are thought to have arisen early after the origin of eukaryotes. To understand the details of distribution and evolution of different PARPs and PAR-regulated pathways we performed a broad analysis of enzymes involved in PAR metabolism across eukaryotic genomes. More than 1900 PARP proteins are present in 249 sequenced eukaryotic species across all six eukaryotic supergroups Opisthokonta, Amoebozoa, Excavata, Chromalveolata, Plantae and Rhizaria. In all these species more than one hundred different types of PARP were found. The majority of these proteins possess the same combination of domains present in human PARP homologues, but many additional distinct domains are also present suggesting novel connections between poly(ADP-ribosyl)ations and different cellular processes (see below).

### 3.1 PARPs in eukaryotes

The increasing number of eukaryotic genomes that have been sequenced reveals that PARP superfamily distribution is wider than previously documented. For the first time it was possible to analyze all six major eukaryotic supergroups, Opisthokonta, Amoebozoa, Plantae, Excavata, Chromalveolata and Rhizaria – because two Rhizaria genomes, from Cercozoa *B. natans* and Foraminifera *Reticulomyxa filosa* have recently been sequenced [52], [58]. Our analyses show that PARP homologues are present in representatives from all major eukaryotic supergroups (Table 1). These findings are in accordance with essentiality of PARP function, which has been already demonstrated in variety of organisms, i.e. mouse, fruit fly, fungus *Aspergillus nidulans* [13], [15], [59]. However, in several sequenced model organisms, PARP homologues are not present suggesting that certain forms of eukaryotic life have adapted to life without PARP signalling. Analyses of recently sequenced genomes revealed that some groups of organisms which were initially proposed to be PARP-deficient include representatives which do in fact possess PARP homologues (red alga *Chondrus crispus*, diatom *Thalassiosira oceanica* and brown alga *Ectocarpus siliculosus*). As already observed in *Chlorella* species [9], we found that the pool of PARP proteins can differ between closely related species in various eukaryotic lineages. For example *T. oceanica* possess at least two PARP family members while the closely related *Thalassiosira pseudonana* is seemingly PARPless.

**Table 1:**
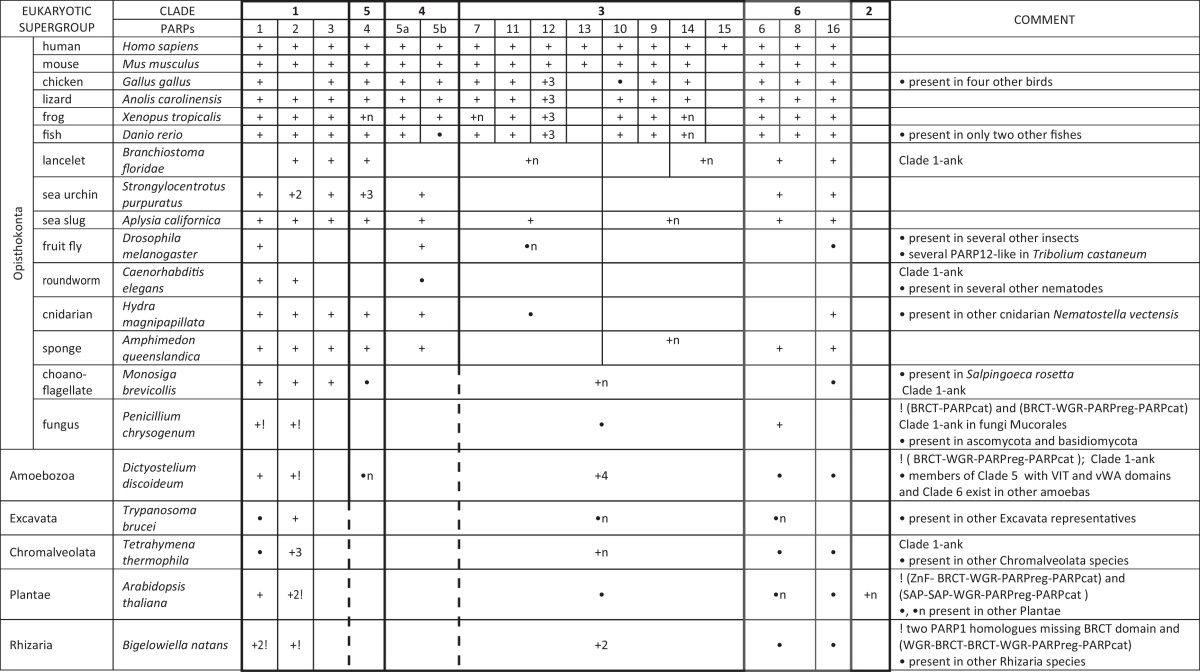
Distribution of PARPs in representative species from six major eukaryotic supergroups.

+ indicates presence of PARP homologue, • indicates presence of PARP homologue in other related species, n indicates presence of more than three PARP homologues, ! indicates comment mark, discontinuous line indicates presence of PARP homologues which group ancestral to clades of common origin. Presence of ankyrin-type Clade 1 PARP (Clade 1-ank) is indicated in the comment section.

On average, the complexity of PARP repertoire increases with the evolutionary level of the species (Table 1). This is most clearly demonstrated in metazoan lineage. In *Sphaeroforma arctica* which is a member of the ichthyosporeans, phylogenetically positioned as a sister group to a clade comprising Choanoflagellata + multicellular animals (Metazoa) lineage, only two PARPs were identified according to currently available genome data. In choanoflagellates, the closest living relatives of the animals, eight types of PARPs were found, while five of them are conserved in human. In sponges (animals that branch off first from common ancestor of all metazoans) nine different types of PARPs are present, with seven different types that can be traced to human. Some animals with accelerated evolution (e.g. nematode *Caenorhabditis elegans* and fruit fly possess only three and two PARPs, respectively) have lost the majority of PARPs present in common metazoan ancestor. Duplication events that preceded the origin of the majority of recent human PARPs likely occurred before the chordate radiation. In nowadays fishes 15 different types of PARPs can be found. The common ancestor of all vertebrates, therefore, probably already had 15 different types of PARPs that are all conserved in human. Finally, the human genome encodes 17 different PARPs (Table 1). Our analysis, based on the amino acids sequence similarity of the catalytic domains, confirmed previously established grouping of 17 human PARPs into five different clades (see supplementary Fig. 1).

Supplementary Fig. S1 related to this article can be found, in the online version, at http://dx.doi.org/10.1016/j.dnarep.2014.05.003.

Fig. S1 Maximum likelihood phylogenetic tree of PARP catalytic domains from representative species. Bootstrap values inferred from 1000 replicates are shown next to the branches. Accession numbers of sequences used are given after species names. The scale bar indicates the genetic distance of the branch lengths.

#### 3.1.1 Clade 1 – DNA repair PARPs

We found orthologues with the same characteristic domain organization to human PARP1 protein in sequenced representatives from five eukaryotic supergroups: Opisthokonta, Amoebozoa, Chromalveolata, Plantae and Excavata. In Rhizaria representative PARP1 homologues are also structurally very similar but lack the BRCT interaction domain. Therefore we can conclude that the last common ancestor of all eukaryotes probably already carried the gene encoding PARP1 with its characteristic domain structure (Fig. 1A). Human PARP2 and PARP3 have a similar domain structure (WGR, PARP regulatory and PARP catalytic domain) with the only difference at their N-terminal ends. Nucleic acid binding domain (NBD), rich in basic amino acids, is present at the N-terminus of human PARP2 protein. Additionally, WWE domain(s) (a putative PAR binding module) is found at the N-terminus of PARP2 homologues in many non-mammalian metazoan representatives (e.g. in bird, frog and fish, Fig. 1A). WGR-PARP regulatory-PARP catalytic domain type of domain organization was proposed to be present in the last common ancestor of all eukaryotes because this type of organization was found in three eukaryotic supergroups [9]. Our analyses support these findings since we have found the same type of domain organization in PARP proteins in five major eukaryotic supergroups, with the exception of Rhizaria. The majority of these proteins contain basic amino acids rich region at N-terminal end which is likely to be involved in nucleic acid binding. We propose that the last common ancestor of all eukaryotes already had two Clade 1 members; one similar to recent human PARP1 and one similar to recent human PARP2. This is in accordance with observation that the functions of PARP1 and PARP2 are complementary but do not fully overlap [60]. PARP1 interacts with PARP2 in single strand break repair and base excision repair pathways and this interaction is essential for the maintenance of genomic stability [13].

In some eukaryotic species, Clade 1 representatives do not have all domains characteristic for certain types of PARP homologue, but rather they display combination of the domains present in all three PARP homologues (i.e. *Acanthamoeba castellanii* in Fig. 1A). Clade 1 also includes plant-type PARP homologue with additional SAP domain(s) at the N-terminus [9]. The SAP domain is the DNA-binding motif predicted to be involved in chromosomal organization. We found this domain sporadically in Clade 1 PARP homologues from representatives of four eukaryotic supergroups: Opisthokonta, Amoebozoa, Chromalveolata and Plantae. This may indicate that the SAP domain also plays an important role in PARP-dependent DNA-damage response, DNA repair and maintenance of genome integrity.

Additionally, a peculiar Clade 1 PARP containing ankyrin repeats (which are always found in tankyrases) at the N-terminus was found in Amoebozoa (e.g. *Dictyostelium discoideum* ADPRT3 protein), Opisthokonta (e.g. PME-5 protein in *C. elegans*), and in Chromalveolata species *Saprolegnia diclina*. PME-5 protein from *C. elegans* has been annotated as tankyrase (see Section 3.1.3 below), but its functional characterization also suggested its role in the DNA damage response [61]. This is in accordance with presumption that all Clade 1 PARP members are involved in DNA repair and genome integrity. However, the DNA-damage response evolved independently in various eukaryotic lineages resulting in different types of Clade 1 pools in various species. For example, PARP2 homologue is present in all analyzed vertebrates, but it cannot be detected in any bird representative with sequenced genome, so we conclude that PARP2 genes are likely lost in birds.

#### 3.1.2 Clade 5 – vault PARPs

It has been already observed that PARP4 homologues are present in the Metazoa and Amoebozoa [9]. PARP4 was originally identified as a protein component of the vault ribonucleoprotein particle in mammals, but the studies in mice showed that the loss of PARP4 protein does not lead to a major defect in the vault structure and function [62]. PARP4 homologues are present in all animals except nematodes and insects (Table 1). Plants and fungi are also missing the PARP4 homologue. This is in accordance with the observation that vaults are missing from the nematode *C. elegans*, fruit fly *Drosophila melanogaster*, fungus *Saccharomyces cerevisiae* and plant *Arabidopsis thaliana* [63]. However, vaults may be present in eukaryotes which do not possess PARP4 homologues, e.g. in *Trypanosoma*, *Leishmania*, *Paramecium* species. Animals usually possess only one PARP4 homologue, but independent duplications occurred in frog, sea urchin and placozoan *Trichoplax adhaerens* (Table 1). In amoeba *D. discoideum*, two proteins phylogenetically close to PARP4, characterized by the absence of VIT and vWA domains were described [9]. We found the third type of PARP4-like protein containing VIT and vWA domains with appended 14-3-3 domain in four other sequenced amoebae; *Dictyostelium fasciculatum*, *D. purpureum*, *Polysphondylium pallidum* and *A. castellanii* as well as in lancelet *Branchiostoma floridae*, sea urchin *Strongylocentrotus purpuratus* and Filasterea *Capsaspora owczarzaki* (Fig. 1B). Proteins with 14-3-3 domain mediate signal transduction, are involved in growth factor signalling and interact with MEK kinases. Notably, it has been shown that PARP1 induces cell death through inhibition of the MEK/ERK pathway in human HeLa cells exposed to the DNA damage [64]. Additional domain at C-terminus is present in some of PARP4-like proteins (e.g. actin, Fig. 1B). In amoeba *A. castellanii* another three types of Clade 5 PARPs were found (Fig. 1B), all with at least one additional domain. In two of them ubiquitin-related domains were detected. A number of links between poly(ADP-ribosyl)ation and ubiquitination pathway have been previously noted [21]. PARP4-like member from choanoflagellate *Salpingoeca rosetta*, as well as homologues from Filasterea *C. owczarzaki* and amoeba *A. castellanii* possess actin or actin-binding domains, indicating connection between poly(ADP-ribosyl)ation and cytoskeletal organization. The observation that overexpression of PARP in *D. melanogaster* disrupts the organization of cytoskeletal F-actin resulting in aberrant cell and tissue morphology [65] may support this connection.

PARP of Clade 5 probably arose in ancestral Unikont (before the separation of Amoebozoa and Opisthokonta). Phylogenetic analysis grouped one Rhizaria PARP within this clade. However this grouping is unlikely to be real, because this protein does not share the characteristic domain structure of PARP4 homologues. From our results we can conclude that the ancestor of choanoflagellates and animals probably possessed only one PARP4 homologue as is still the case in most animals and in filose amoeboid *C. owczarzaki*, which belongs to unicellular lineage that forms a sister-group to multicellular animals. However, an expansion of Clade 5 PARP proteins happened in Amoebozoa.

#### 3.1.3 Clade 4 – tankyrases

Tankyrases with the same domain structure as human homologues (Fig. 1C) were described only in animals with bilateral symmetry [9]. However, we found partial tankyrase in cnidarian *Hydra magnipapillata* (XP_00215846) and in sponge *Carteriospongia foliascens* (GO083148), as well as miscRNA coding for tankyrase in sponge *Amphimedon queenslandica* (XR_131689). Presence of tankyrase in sponges, which branch off first from the common ancestor of all animals, indicates that tankyrase appearance correlates with the appearance of metazoan multicellularity. Unusual grouping of *D. discoideum* pARTf (see supplementary Fig. 1) was also previously observed [9].

It was proposed that the duplication event generating two tankyrases appeared sometime after the separation of the amphibians [9]. However, our data suggest that the duplication event related to the origin of the more recent tankyrase 2 appeared probably sometime before the separation of fishes, because in fish *Oreochromis niloticus* both tankyrases are present (XP_003445711, XP_003449482). In the nematode *C. elegans* and lancelet *B. floridae* human-type tankyrase is not present (Table 1). However, they both possess PARP protein with ankyrins belonging to Clade 1 (see Section 3.1.1). In nematode *C. elegans* this type of protein (named PME-5) was initially identified as tankyrase [61], [66]. However, cellular localization of PME-5 differs from the localization of human tankyrases and an additional role of this protein in response to DNA damage has been proposed. Interestingly, in a different nematode *Brugia malayi* human-type tankyrase were found, and the Clade 1 PARP protein with ankyrins is missing. This indicates that these two proteins have at least some overlapping functions and can replace each other. However, combination of ankyrin repeats and PARP catalytic domain is not exclusive to proteins from *C. elegans* and *B. floridae*, and are found in other eukaryotic PARPs including excavate *Naegleria gruberi*, choanoflagellate *Monosiga brevicollis* and *Dictyostelium* species, which suggest that this combination of domain arose independently several times in eukaryotic evolution.

#### 3.1.4 Clade 6 – (PARP6, 8 and 16)

According to phylogenetic analysis, PARP6 and PARP8 probably arose in fishes. In all major eukaryotic supergroups, an ancestral type-PARP6/8 homologue also exists. A truncated catalytic-like domain has been proposed to lie in the region preceding the PARP domain [60], [67]. In this region we identified cysteine rich region (CRR in Fig. 1D) with a putative ZnF motif CX_2_CX_9–19_CX_4_C which can be found in PARP6/8 homologues from representatives of all eukaryotic supergroups.

PARP6/8 homologues from Ascomycota, Basidiomycota, moss *Physcomitrella patens* and green algae *Chlamydomonas reinhardtii* have additional UBCc domain. This domain is a part of the catalytic domain of ubiquitin-conjugating enzyme E2 [68], and also supports the connection between PARP proteins and ubiquitination. In amoeba *A. castellanii* and excavate *Trichomonas vaginalis* PARP6/8 homologue has additional UBA (ubiquitin-associated) domain (Fig. 1D). UBA domains were found in diverse proteins involved in numerous cell processes including the ubiquitin/proteasome pathway [69]. Another link with ubiquitination was found in PARP6/8 homologue from Rhizaria *R. filosa* which contains appended RWD domain (a domain related to the UBCc) [70] (Fig. 1D).

The majority of metazoans (except animals with accelerated evolution – nematodes and fruit fly) possess a PARP16 homologue (Table 1). PARP16 proteins are sporadically found in many non-metazoan species (e.g. choanoflagellate *S. rosetta*, amoeba *A. castellanii*, chromalveolate *E. siliculosus*, Rhizaria *R. filosa*, green algae *Chlorella variabilis* and *Volvox carteri*), but these proteins always lack the α-helical domain characteristic of the metazoan PARP16. Furthermore, in some insects (honey bee *Apis mellifera*, bumble bees and some ants) PARP16 homologue has the two C-terminal Cyclin domains that replace a canonical C-terminal PARP16 transmembrane domain.

We found Clade 6 representatives in all six major eukaryotic supergroups (Table 1). Our analyses show that last common ancestor of all eukaryotes possessed two Clade 6 members. Clade 6 representatives are biochemically and physiologically poorly characterized, but presence in the last common ancestor of all eukaryotes indicates its importance in eukaryotic evolution. As already mentioned, PARP16 homologue containing both characteristic domains was found in five out of six eukaryotic supergroups. The first ancestral type of Clade 6 proteins was probably similar to recent human PARP16. It was shown that the human PARP16 is upregulated during endoplasmic reticulum stress and is required for the unfolded protein response [71], [72]. These data may indicate a general importance of PARP16 in stress response early in eukaryotic evolution. We propose that the second ancestral type of Clade 6 proteins was PARP6/8 homologue, and likely consisted of CX_2_CX_9–19_CX_4_C motif and PARP domain. Appropriately, our analyses show that the PARP6/8 homologues are also widely distributed in eukaryotes. The presence of additional ubiquitination-related domains in fungal, Excavata, amoebae and bryophyte representatives may indicate an ancestral link of PARP6/8 to ubiquitination pathways. PARP8 knockdown in human cells resulted in cell morphology defects and the most pronounced decrease in cell viability of all PARPs [72], which may indicate its essentiality and explain its wide distribution.

#### 3.1.5 Clade 3

Clade 3 encompasses most heterogeneous PARPs, in terms of their functions and domain structure. Presence of Clade 3 representatives in all eukaryotic supergroups indicates their importance early in eukaryotic evolution.

PARP7, 12 and 13 homologues possess ZnF_C3H1 which can bind to RNA, while PARP11 likely arose after a duplication event during in which this ZnF was lost. Human PARP7 and PARP11 homologues are confirmed only in vertebrates, and they likely arose sometime before the separation of fishes. Phylogenetic analysis showed that PARP13 arose from PARP12 after a duplication event that appears to have occurred sometime after the separation of mammals. Two proteins in cnidarian *Nematostella vectensis*, a few in insect *Tribolium castaneum* and two in lancelet *B. floridae* group together and probably represent an ancestor of the PARP7/11/12/13 branch of Clade 3 proteins (Table 1). Cnidarian and insect proteins contain only PARP domain (and WWE sporadically). From several ancestral PARP7/11/12/13 present in lancelet *B. floridae*, two acquired ZnF_C3H1 but only one has domains corresponding to vertebrate homologues (Fig. 1E). The PARP7/11/12/13 ancestor probably arose sometime before the separation of cnidarians. It was most similar to recent PARP12 and is therefore named PARP12-like. PARP12-like protein exhibits expansion in different lineages through duplications (e.g. in frog and fishes, Table 1). One duplication event eventually led to PARP13 appearance in mammals. In sponge *A. queenslandica* one protein that has been annotated in the databases as PARP12-like is placed by our phylogenetic analysis as ancestral-type representative of all Clade 3 (see supplementary Fig. 1). This protein has N-terminal transmembrane domain followed by PARP domain. Overall, a general RNA binding function could be predicted for the C3H1 ZnF type PARPs (which includes human PARP7/12/13 proteins), however the precise function of their PARP-catalytic domain remains largely unknown [73]. The most studied representative is mammalian PARP13 (ZC3HAV1), which has been shown to act as a ZnF antiviral protein [74]. Also PARP13 was shown to be critical for microRNA silencing [75]. Susceptibility to multiple sclerosis is also linked to PARP13 [76]. Recently, it was demonstrated that some other Clade 3 members PARP7, PARP10 and PARP12 may also function as important regulators of cellular translation and virus replication [77].

PARP10 homologues are present only in vertebrates, and function as mono(ADP-ribosyl) transferases [23]. Human PARP10 homologues contain an RRM (RNA recognition motif) domain followed by three UIM domains (the ubiquitin interacting motif) and PARP domain. In frog *Xenopus tropicalis* we found four UIM domains whilst there was absence of the RRM domain. Interestingly, in *Danio rerio* and *Gallus gallus* additional WWE domain that precedes PARP domain was found, while this region in mammalian and frog protein is missing (Fig. 1E). PARP10 activity probably controls a number of different signalling processes [28]. Human PARP10 localizes primarily in cytoplasm, but it was shown also to interact with the proto-oncoprotein MYC [78]. PARP10 recognizes ubiquitin with its UIM motifs, and modulates the NF-κB signalling pathway [79].

Key functional domain, common for human PARP9, PARP14 and PARP15 proteins is a macrodomain. The macrodomain PARP ancestor probably arose early in Unikonts (predecessors of Amebozoa, Opistokonta and Apusozoa) evolution. It is most similar to the recent PARP14 homologue and is therefore named PARP14-like. PARP14-like proteins are found to be frequently duplicated (Table 1). Even in vertebrates PARP14 proteins are readily found duplicated and one of this duplication is actually PARP15. Phylogenetic analysis showed that PARP15 arose from PARP14 after duplication event that appears to have occurred sometime after the separation of mammals. It is striking that PARP14 duplications are found often very near on the chromosome (e.g. in lancelet *B. floridae* at least four PARP14-like proteins are grouped together on the same chromosome). In basal metazoans (placozoan and sponge) we also found frequent PARP14-like duplications. In sponge *A. queenslandica*, six different PARP14-like proteins are found and they all have at least one macrodomain (some two or three of them), some have WWE, some RRM and one has an additional Radial spoke3 domain (domain important in cilia and flagella motility). Clade 3 proteins from representatives of choanoflagelate and Amoebozoa also possess characteristic domains (macrodomain, RRM, WWE and PARP domain). In *Dictyostelium* species an additional N-terminal U-box, a common domain in ubiquitination [80] was found (Fig. 1E). In Chromalveolata *Paramecium tetraurelia* Clade 3 protein containing macrodomain followed by PARP domain was found (Fig. 1E), which may suggests that PARP proteins acquired the macrodomain at least twice in eukaryotic evolution.

Human PARP9 (BAL1) orthologues are found only in vertebrates. They possess two macrodomains and a PARP domain which is apparently ADP-ribosyl transferase inactive [81]. Human PARP9 recognizes PAR via its macrodomains and has an unclarified role in the DNA damage response [26].

Clade 3 is proposed to be somewhat artificial and the domain structures outside the PARP catalytic domain are heterogeneous. Accordingly, Clade 3-type proteins from Rhizaria species differs among themselves and contain domains not previously found in any other Clade 3 homologue (ZnF-RBZ, H_lectin, transmembrane domain). Representatives from all major eukaryotic supergroups contain PARP Clade 3 member (Table 1). Since Clade 3 proteins are very heterogeneous both in structure and function, it is hard to presume what domain structure of ancestral-type of protein was present and what functions were encompassed. Recently it has been shown that PARP15, together with PARP5a, PARP12, PARP13 and PARP14, function in the assembly of cytoplasmic stress granules, cellular macrostructures that aggregate translationally stalled mRNA–protein complexes [75]. Since function of stress granules encompasses both ZnF_C3H1 and macrodomain Clade 3 members this may indicate a possible function of the ancestral Clade 3 protein.

#### 3.1.6 Clade 2 – plant PARPs

Land plants are characterized by a distinct group of PARP-like proteins, the SRO family that forms a plant-specific Clade 2. Representatives of the SRO family from *A. thaliana* are RCD1 (radical-induced Cell Death 1) and SRO1-5 (similar to RCD1) proteins. RCD1 protein exhibits WWE-PARP domain-RST domain organization, while SRO proteins are missing N-terminal WWE domain. All members of this protein family contain plant-specific RST domain which is required for interaction with multiple plant transcription factors [20]. Additional domains are very rare, for instance the transmembrane domain at the C-end of *Oryza sativa* Clade 2 PARP (Fig. 1F). The PARP specific signature is not well conserved within this protein family. Biochemical analysis of *A. thaliana* RCD1 suggested that SROs do not possess ADP-ribosyl transferase activity [19]. The function of SROs is nevertheless critical for plants since RCD1 and SRO1 proteins appear to be essential for proper development in *A. thaliana* and several SRO family members have been implicated in the stress response [82].

Overall, the distribution of PARPs among eukaryotes indicates the importance of poly(ADPribosyl)ation in ancestor of all eukaryotes and their involvement in wide variety of pathways.

### 3.2 PARGs in eukaryotes

More than 300 canonical PARGs are present in 150 eukaryotic species across all six eukaryotic supergroups. Duplications and multiplications of PARG genes are quite common, and are observed in representatives from all of these major eukaryotic supergroups (Table 2). For example, in the plant *A. thaliana*, different PARGs show different responses to microbe-associated molecular patterns, while in the nematode *C. elegans*, different PARGs show different localization and one PARG is predominantly expressed [83], [84]. BactPARGs are found in representatives from all eukaryotic supergroups, except Plantae, but are substantially less distributed among eukaryotes than the canonical type. The number of bactPARG genes in eukaryote genomes varies from one to up to seven, as seen in Excavata *N. gruberi*. However, these proteins vary at PARG signature sequence, and only three of them have fully conserved GGG-X_6–8_-QEE catalytic motif. Other proteins have only partially conserved PARG signature (e.g. GGA/H-X_7_-QEE). Although we included all this PARG proteins in Table 2, we can only speculate about their functionality. Some species have both types of PARGs, for example, filamentous fungi Ascomycota (e.g. *Podospora anserina*, *Penicillium chrysogenum*, *Nectria haematococca*). Representatives of other filamentous fungi Basidiomycota and Ascomycota (e.g. *Thielavia terrrestris*) and some other eukaryotes do not possess canonical type, but in their genomes gene encoding bactPARG is present. Besides yeasts, in several other genomes we could not find an obvious PARG homologue (e.g. Mucorales *Mucor circinelloides* and *Rhizopus delemar*, Chromalveolata *S. diclina* and *E. siliculosus*, Rhodophyta *C. crispus*, Chlorophyta *C. variabilis*, *C. reinhardtii* and *V. carteri*).

**Table 2:** Distribution of PAR erasers in representative species from six major eukaryotic supergroups.

Eukaryotic supergroup	Organism	PARG/bactPARG	ARH3	MacroD1	MacroD2	TARG1	PBZ
Opisthokonta							
Human	*Homo sapiens*	+	+	+	+	+	+
Mouse	*Mus musculus*	+	+	+2	+	+	+
Chicken	*Gallus gallus*	+	+		+	+	+
Lizard	*Anolis carolinensis*	+	+	+	+	+	+
Frog	*Xenopus tropicalis*	+	+2	+	+2		+
Fish	*Danio rerio*	+2	+	+	+	+3	+
Lancelet	*Branchiostoma floridae*	+2	+	+			+
Sea urchin	*Strongylocentrotus purpuratus*	+3	+	+		+	+
Sea slug	*Aplysia californica*	+2	+	+			+
Fruit fly	*Drosophila melanogaster*	+				+2	+
Roundworm	*Caenorhabditis elegans*	+2		+			+
Cnidarian	*Hydra magnipapillata*	+	+			+	
Sponge	*Amphimedon queenslandica*	+3	+	+2		+	+
Choanoflagellate	*Monosiga brevicollis*	+/+					+
Fungus	*Penicillium chrysogenum*	+/+2	+	+			

Amoebozoa	*Dictyostelium discoideum*	+		+			+
Excavata	*Trypanosoma brucei*	+		+			+
Chromalveolata	*Tetrahymena thermophila*	+7	+				+
Plantae	*Arabidopsis thaliana*	+2		+			+
Rhizaria	*Bigelowiella natans*	+2/+3	+4	+			

+ indicates presence of eraser homologue; number indicates presence of several eraser homologues.

**Fig. 2:**
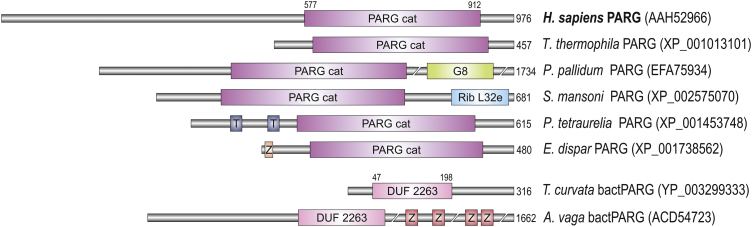
Domain architecture of PARG and bactPARG enzymes. Abbreviations of domain names are retrieved from SMART/Pfam databases and indicated in figure. Domains which are not retrieved from SMART/Pfam databases: Z (red and pink), represent Zn finger motifs, poly(ADP-ribose)-binding Zn finger (PBZ) in *A. vaga* and polyA-RNA-binding Nab2-type of ZnF in *E. dispar*, respectively.

In only a few species, PARG proteins with additional domains distinct from human proteins were found. In rotifer *Adineta vaga*, several repeats of PBZ zinc fingers were previously identified [18]. PolyA RNA-binding Nab2 type of Zinc finger [85] is found in *Entamoeba dispar* PARG (XP_001738562). In amoeba *P. pallidum*, ciliate *P. tetraurelia*, and trematode *Schistosoma mansoni*, G8, transmembrane and Ribosomal_L32e domains were found, respectively (Fig. 2).

**Fig. 3:**
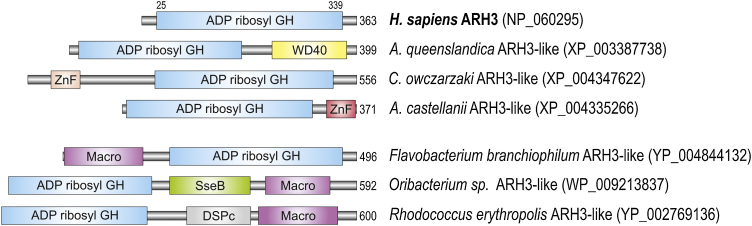
Domain architecture of human ARH3 and ARH3-like proteins from representative species. Abbreviations of domain names are retrieved from SMART/Pfam databases and indicated in figure. Shortened names include: ZnF (red and pink), ZnF-RBZ in *A. castellanii* and Zf-UBP in *C. owczarzaki* ARH3-like, respectively.

Another enzyme that possesses PARG-like activity, ARH3, shows no structural or sequence similarity to PARG and does not follow the PARPs distribution. The ARH protein family is widely distributed among all three domains of life. The ancestral type of ARH3 probably arose before metazoan appearance. However, in amoeba, Filasterea and sponge ARH3 holomogues, additional domains were found (zf-RanBP, zf-UBP and WD40, respectively; Fig. 3). The majority of other ARHs from six eukaryotic supergroups show higher similarity to ARH3 rather than to ARH1/2 (which do not display activity against PAR), and have no additional domains. It is difficult to determine whether ancestral types of proteins have similar biochemical properties and function as human homologues and when the true ARH3 homologue arose.

### 3.3 MacroD1, MacroD2 and TARG1 in eukaryotes

Neither PARG nor ARH3 are capable of cleaving the ester bond between the proximal ADP-ribose unit of target proteins. Human MacroD1, MacroD2 and TARG1 (C6orf130) are the only enzymes described so far that can cleave the ADPr directly linked on glutamate and aspartate [6], [46], [47]. MacroD proteins are found in representatives from all major supergroups of eukaryotes (Table 2) [86]. However, some species from Opisthokonta (e.g. cnidarian *H. magnipapillata*, fruit fly, choanoflagellates, fungus *N. heamatoccoca*) Excavata (e.g. *T. vaginalis*) and Chromalveolata (e.g. *P. tetraurelia*) have lost the MacroD gene from their genomes. MacroD1 and MacroD2 originate from a duplication event that occurred in the last common ancestor of vertebrates. MacroD1 was not found in any sequenced birds, indicating a possible loss of this gene in this lineage.

TARG1 is less distributed among eukaryotes. In vertebrates TARGs are readily found, but their homologues were found only in some representatives of other Opisthokonta groups. In Fungi (e.g. in some Mucorales, Basidiomycota and Ascomycota representatives) and invertebrates we found them only occasionally (e.g. in sponge, sea urchin and fruit fly (Table 2)). Sporadic distribution among fungus lineage, as well as absence of TARG1 homologues in Choanoflagellata and Filasterea species indicates lots of secondary losses. However, at least one de-mono(ADP-ribosyl)ating type of enzyme is readily retained in the organisms, which suggests the overlapping functions for TARG and MacroD proteins. For example, in fruit fly *D. melanogaster* and other flies only the TARG1 homologues are found. On the other hand, in honey bee *A. melifera* the TARG homologues were lost, while a MacroD protein is retained instead (Table 2).

### 3.4 PBZ

PBZ is a conserved PAR-binding module restricted only to eukaryotic proteins and coevolved strictly with PARPs [50], therefore we used this module as a marker for presence of PAR metabolism. In mammals only three proteins have PBZ domains (APLF, CHFR and SNM1A). In amoeba *D. discoideum* PBZ domain is more widespread and appears to be limited to proteins involved in the DNA damage response including PARPs [50], [87]. PBZ in combination with PARP catalytic domain was previously found in Amoebozoa *Entamoeba histolytica* [50]. Moreover, this combination was found in Filastera and in metazoan lineage (e.g. in *C. owczarzaki* and *B. lancelet*). PBZ was primarily found in eukaryotic proteins involved in the DNA damage response, often in association with other domains known to occur in PARPs (BRCT, RRM, and RING domains). Overall our study shows that the PBZ distribution correlates excellently with the distribution of PARPs, except in Fungi in which this motif could not be found (Table 2).

### 3.5 Prokaryotes

According to our analysis, 28 PARP homologues can be found in 27 bacterial species, which belong to six different phyla of the Bacteria domain. Since PARP homologues were identified in minor subset of total 30 bacterial phyla and were not found widespread within each phylum, we can conclude that PARP proteins in bacteria sporadically acquired their PARP genes through horizontal gene transfer. Only one bacterium, *Microscilla marina*, possesses two PARP genes in its genome. Most of bacterial PARPs contain only two domains (WGR and PARP domain). Seven bacterial PARPs have additional PARP regulatory domain, and seven are composed solely of the PARP domain. Majority of bacterial PARPs are most similar to the DNA repair-specific Clade 1 PARP homologues. The catalytic triad H-Y-E essential for poly(ADP-ribosyl)ating activity of PARPs is conserved in majority (27 out of 28) of analyzed bacterial PARPs. In the remaining PARP only third amino acid residue of catalytic triad is not conserved. This protein is most similar to PARP1 homologues indicating that the mutation which may have changed its ADP-ribosylation function (poly vs mono(ADP-ribosyl)ation) occurred after horizontal gene transfer. A recent study showed that PARP from bacterium *Herpetosiphon aurantiacus*, with conserved catalytic triad, possesses the same characteristics as human PARP1 enzyme; it requires DNA for its activation and is able to synthesize long chains of PAR [18].

Distribution of PAR erasers was checked in bacteria representatives which encode PARP and have fully sequenced genome (Table 3). Presence of PARG genes does not correlate strongly with PARPs in Bacteria. According to our analyses more than 150 bacterial species, distributed in thirteen phyla, contain PARG homologue(s). However, only seven of PARP-encoding fully sequenced bacterial genomes possess also PARG-encoding gene(s). The fact that the majority of bacterial genomes that possess PARG gene do not possess PARP gene, raises the question of alternative PARG function(s) in these bacteria. In some cases it may serve to recycle PAR from the environment or as a defence mechanism against ADP-ribosylating toxins [18]. Also, it cannot be excluded that some bacteria possess a highly divergent or unrelated form of PARP [18]. The fact that the *Deinococcus radiodurans* PARG homologue is one of the most induced genes after DNA damage, suggests that PAR metabolism in bacteria might be related to DNA damage response [88]. In only two PARGless bacterial species, that possess PARP in their genomes, potential alternative PAR eraser (ARH3-like homologue) was found (Table 3). Almost all of these bacteria possess at least one MacroD-like or TARG1-like protein in their genome which could possibly provide an alternative mechanism for the reversing of protein ADP-ribosylation. In three sequenced bacterial species with PARPs, none of the ADP-ribosylation-removing enzymes were detected. We cannot exclude the possibility that as yet unidentified analogues of PAR erasers are present in some bacteria. Some bacterial species possess proteins with combination of ARH3-like and MacroD1/2-like domains (Fig. 3). Interestingly, all amino acids essential for function of both domains are conserved, which may indicate that these bacteria arrange a functional “all in one” PAR eraser.

**Table 3:** Distribution of enzymes involved in PAR metabolism in representative bacterial species.

Phylum	Bacterium	PARP	PARG	ARH3-like	MacroD-like	TARG1-like
Firmicutes	*Bacillus thuringiensis*	+		+	+	+
	*Paenibacillus polymyxa*	+	+	+3	+	+
	*Clostridium citroniae*^a^	+	+	+2	+2	+
	*Butyrivibrio proteoclasticus*	+	+	+	+	+2
	*Eubacterium rectale*	+	+2		+5	

Bacteroidetes	*Fibrella aestuarina*	+		+2	+	
	*Microscilla marina*	+2	+2	+8	+	+
	*Fibrisoma limi*	+			+	
	*Spirosoma linguale*	+				
	*Flexibacter litoralis*	+	+2	+2	+	+

Actinobacteria	*Mycobacterium abscessus*	+				
	*Microbacterium maritypicum*	+				

Proteobacteria	*Vibrio fluvialis*^a^	+		+	+	+
	*Plesiocystis pacifica*^a^	+	+	+3	+	

Cyanobacteria	*Stanieria cyanosphaera*	+				+
Chloroflexi	*Herpetosiphon aurantiacus*	+	+	+3	+2	+

a Bacterial species with draft sequenced genome; number indicates presence of several protein homologues.

### 3.6 Archaea

Although there are no detectable PARP genes in archaeal genomes, endogenous ADP-ribosylation activity has been detected in the archaeon *S. solfataricus*. A protein from *S. solfataricus* (PARPSso) has been partially purified and showed to possess an oligo(ADP-ribosyl) transferase activity with non-specific DNA-binding activity [39]. Identified target proteins of PARPSso are PARPSso itself and a 7-kDa protein (Sso7) which replaces histone-like proteins in sulphur-dependent extremophiles [89].

Genes encoding PARG proteins have not been found in archaeal genomes, but many archaeal genomes encode other macrodomain proteins. The best studied archaeal macrodomain protein is Af1521 from *Archaeoglobus fulgidus* [90]. Af1521 is capable of binding both ADPr and PAR and has enzymatic activity capable of hydrolysing mono(ADP-ribosyl)ated protein substrates [26], [46].

### 3.7 Viruses

We have identified PARP genes in four genomes of dsDNA viruses (Aeromonas phage Aeh1, Anticarsia gemmatalis nucleopolyhedrovirus, Invertebrate iridescent virus 6 and Cellulophaga phage phi4:1). In all cases the genes are probably gained from their hosts. The catalytic triad H-Y-E is fully conserved in three of these viral PARPs, and only one has Asp instead of Glu, which may suggest that these PARPs are active ADP-ribosyl transferases. Some viruses use PAR metabolism for their replication. For example, Herpes Simplex Virus and Epstein–Barr Virus require PARP activity for efficient replication [91], [92].

In three other dsDNA viruses (Bacillus phage G, *Pandoravirus dulcis* and *Pandoravirus salinus*) bactPARG homologues were found, all with a fully conserved PARG signature. In addition, eight ARH3-like proteins were found, predominantly in dsDNA viruses with conserved majority of amino acid residues essential for activity of human ARH3.

The most distributed domain involved in PAR metabolism found in viral genomes is the macrodomain. This domain is found in dsDNA viruses, but also in ssRNA positive-strand viruses, and is usually found as a part of larger proteins which contain additional domains. Most of the analyzed macrodomains possess amino acid residues essential for human MacroD1/D2. Although TARG1-like homologues were identified in numerous viral genomes, only a few of them have conserved all amino acid residues essential for TARG1 activity. Macrodomains in viruses are the most studied members of PAR metabolism family, with several crystal structures solved [93], [94]. It has been demonstrated that macrodomains derived from several viral proteins that interact with both PAR and ADPr in vitro [48] and the macrodomain from Sindbis virus nsP3 protein is important for replication in neurons and neurovirulence in mice [95]. Biochemical, structural and phylogenetic evidences suggest that viral and cellular macrodomains are strongly related, and have all predispositions to act as a glycohydrolase of terminal ADPr on mono(ADP-ribosyl)ated substrates.

The distribution of proteins involved in PAR metabolism in viruses suggests frequent viral interaction with cellular PAR pathways. It was demonstrated that Sindbis virus nsP3 protein interacts with PARP1 and activates PARP1 in neuronal cells while HSV-1 infection actively alters the fine-tuned balance in cellular PAR metabolism [96], [97]. The presence of cellular antiviral PARPs and PAR erasers in viral genomes suggest intensive host–virus coevolution of PAR metabolism.

## 4 Conclusions

All components essential for PAR metabolisms were present in the common ancestor of all eukaryotes, which suggest the importance of poly(ADP-ribosyl)ation in cell physiology of the ancestral eukaryote. The last common ancestor of eukaryotes possessed at least five types of PARP proteins. Two of them were involved in DNA damage response and genome integrity. They are maintained in many recent eukaryotes and correspond to human PARP1 and PARP2 homologues. One of the ancestral PARPs was similar to recent PARP16 and was possibly involved in the early stress response. The other ancestral PARP type corresponds to recent PARP6/8. The presence of ubiquitination-related domains in PARP6/8 homologues from representatives of evolutionary distinct eukaryotic supergroups indicates connection of that ancestral PARP type with ubiquitination. This demonstrates that the crosstalk between PARPs and ubiquitination systems is even more widespread than previously thought (altogether, our analyses demonstrate that representatives of PARP proteins from all six clades are linked to ubiquitination). The last PARP type present in last common ancestor of all eukaryotes was founder of recent heterogeneous Clade 3 characterized by representatives with various functional domains.

Our insight into the distribution of enzymes involved in PAR metabolism among eukaryotes also provides a basis for the selection of model organisms with an adequate genetic background appropriate for investigation of specific human PARP proteins. For example, the PARP2 homologue cannot be detected in any sequenced bird genome and consequently this protein cannot be specifically studied in these organisms. However, PARP1 and PARP2 redundancy in humans perhaps makes birds good model organisms for studying PARP's function in the early sensing and signalling of DNA single strand breaks. The fruit fly *D. melanogaster* is potentially another good model for studying cellular roles of PARP1 as it is one of the only few organisms with just one Clade 1 member and its PARP1 orthologue has the identical domain composition as the human orthologue. Furthermore, *D. melanogaster* and fish *D. rerio* possess only one tankyrase which could be advantageous for studying tankyrase function. Nematode *C. elegans* is an established model for studying Clade 1 representatives including the ankyrin-type (see Section 3.1.3 above), although the conservation of the PAR-related human proteins is not ideal in this organism. *D. discoideum* is potentially useful model organism for studying DNA damage response PARPs [17]. Conservation of PARP16 suggests that the PARP could be studied in a number of different model organisms.

## Conflict of interest

The authors declare no competing financial interests.
