# Supplementary material for: Distribution of protein poly(ADP-ribosyl)ation systems across all domains of life
Source: DNA Repair (Amst). 2014 Nov;23:4–16. doi: 10.1016/j.dnarep.2014.05.003 (PMC4245714; doi:10.1016/j.dnarep.2014.05.003)
Supplement: Fig. S1 — Maximum likelihood phylogenetic tree of PARP catalytic domains from representative species. Bootstrap values inferred from 1000 replicates are shown next to the branches. Accession numbers of sequences used are given after species names. The scale bar indicates the genetic distance of the branch lengths. [file mmc1.pdf]

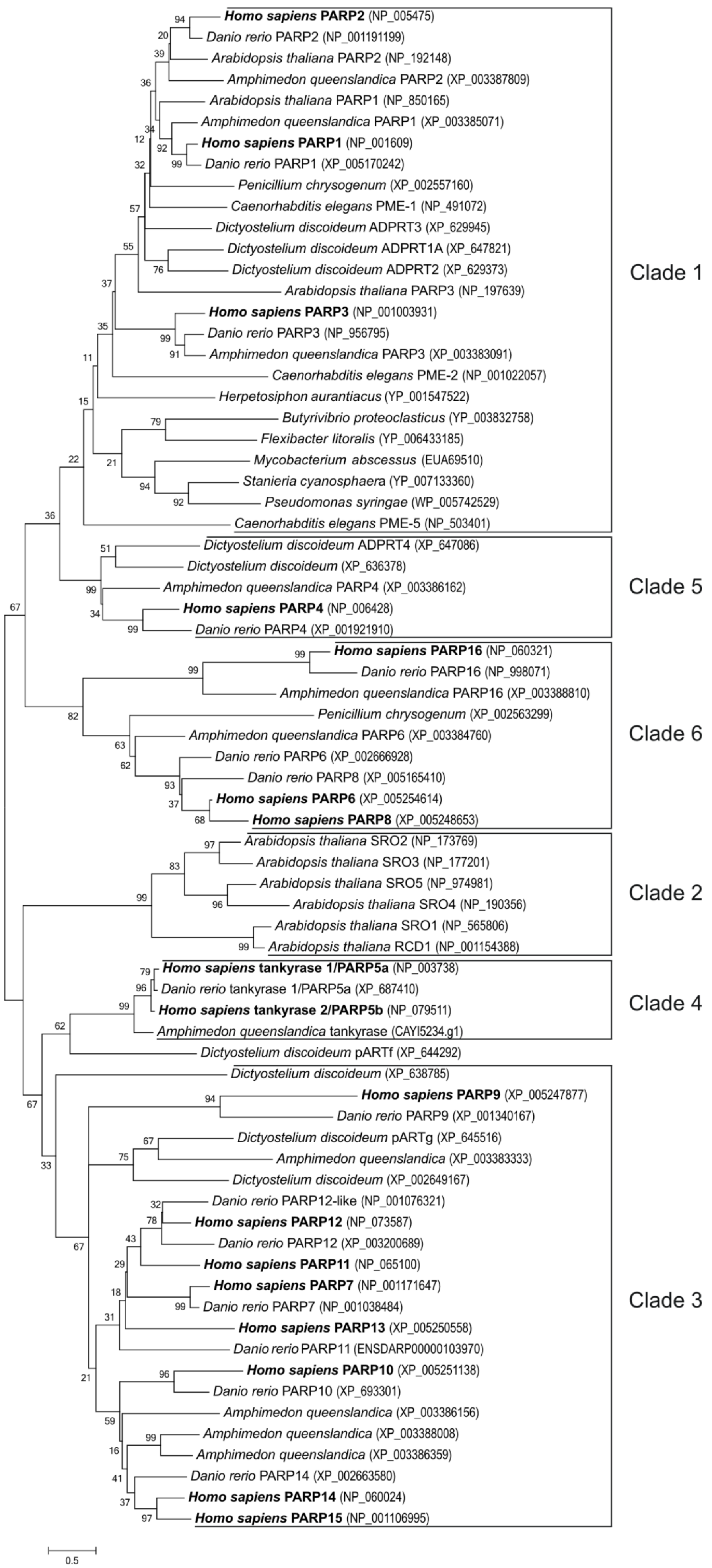

Figure 1. Maximum likelihood phylogenetic tree of PARP catalytic domains from representative species. Bootstrap values inferred from 1000 replicates are shown next to the branches. Accession numbers of sequences used are given after species names. The scale bar indicates the genetic distance of the branch lengths.
